# Mupirocin resistance among MRSA surveillance isolates from neonatal intensive care unit under 10 year-search and destroy policy compared to

**DOI:** 10.1186/1753-6561-5-S6-P171

**Published:** 2011-06-29

**Authors:** M-N Kim, H Kim, D An, M Ji, M Lee, A-R Kim, SJ Lee

**Affiliations:** 1 Laboratory Medicine, Asan Medical Center, Seoul, Korea, Republic Of; 2 Medical Education, Chungbuk National University, Chungju, Korea, Republic Of

## Introduction / objectives

One of major threatening of search and destroy of MRSA is rising mupirocin resistance. In a 2,200-bed tertiary care hospital, only 38-bed neonatal intensive care unit (NICU) was under active surveillance and decolonization of MRSA colonizers for last 10 years. Mupirocin resistances of MRSA surveillance isolates from NICU and clinical isolates of *Staphylococcus aureus* from the institute were compared.

## Methods

From October 2008 to September 9, all MRSA surveillance isolates from NICU mupirocin MICs were determined by E-test (AB Biodisk, Solna, Sweden). From December 2009 to March 2010, 500 clinical isolates of *S. aureus* were consecutively collected by the first isolate per a patient and their MICs of mupirocin were measured using MicroScan Pos Breakpoint Combo Panel Type28 (Siemens, USA) and MIC of resistant isolates were confirmed by E-test. All mupirocin resistant isolates were submitted to PCR for *mupA*.

## Results

70 (10.8%) of 648 patients were MRSA-colonized in NICU. 11 (15.7%) of 70 MRSA surveillance isolates were mupirocin-resistant with high-level MIC. Clinical isolates comprised 315 MRSA and 185 methicillin-susceptible *S. aurues* (MSSA). 39 (7.8%) including 30 (9.5%) MRSA and 9 (4.9%) MSSA were mupirocin-resistant, but only 8 MRSA (26.7%) and 7 (77.8%) MSSA had high-level MIC. All high-level resistant isolates and no low-level resistant one were *mupA*-positive.

## Conclusion

Mupirocin high-level resistance of MRSA surveillance isolates under the search-and-destroy condition was much higher than clinical isolates of MRSA or MSSA. MRSA decolonization requires caution and monitoring of rising mupirocin-resistance.

## Disclosure of interest

None declared.

